# Effects of carriers for oils in compound feeds on growth performance, nutrient digestibility, and gut microbiota in broiler chickens

**DOI:** 10.1016/j.psj.2024.103803

**Published:** 2024-05-03

**Authors:** Florian Quinger, Julia Kern, Astrid Bosse, Jana Seifert, Markus Rodehutscord, Wolfgang Siegert

**Affiliations:** ⁎ Institute of Animal Science, University of Hohenheim, 70599 Stuttgart, Germany; † J. Rettenmaier & Söhne GmbH + Co KG, 73494 Rosenberg, Germany; ‡ Hohenheim Center for Livestock Microbiome Research (HoLMiR), University of Hohenheim, 70599 Stuttgart, Germany; § Department of Animal Sciences, University of Göttingen, 37077 Göttingen, Germany

**Keywords:** broiler chickens, oil carrier, performance, digestibility, microbiota

## Abstract

Carrier materials for oils in compound feeds may be used in animal nutrition to supply liquid feed additives. However, implications of such carriers for the digestibility of the contained oil are unknown. This study investigated the potential of oil carriers in compound feed and their effect on performance, metabolizable energy, fatty acid (**FA**) retention, amino acid (**AA**) digestibility, and gut microbiota in broiler chickens. Six experimental diets were formulated following a 2 × 3 factorial arrangement with 20 g/kg or 40 g/kg of rapeseed oil supplied with no carrier or bound in a silica-based (**SC**) or lignocellulose-based (**LC**) carrier in a 1:1 mass ratio. The diets were assigned to 48 metabolism units with 15 animals each based on a randomized complete block design and fed from d 18 to 28 of the trial. Total excreta were collected from d 24 to 27 and used to determine total tract retention (**TTR**) of FA and MEn. On d 28, AA digestibility both by the distal half of the jejunum and the distal half of the ileum was determined, and microbiota of ileal and cecal digesta was analyzed using 16S ribosomal RNA sequencing. There were significant interactions for ADG, ADFI, the gain:feed ratio (**G:F**), MEn, and the TTR of crude fat and most fatty acids (*P* ≤ 0.046) except for C18, C18:2, and C22:0. Addition of SC decreased ADG, ADFI, and G:F (*P* < 0.001), while LC at 40 g/kg oil inclusion increased G:F and MEn (*P* < 0.001) for both inclusion levels. The TTR of crude fat and the FA C18:1, C18:2, C18:3, and C22:0 was increased by the addition of SC (*P* ≤ 0.016), while LC increased the TTR of the FA C18:1 and C18:2 as well as the TTR of C18:3 at 20 g/kg oil inclusion (*P* ≤ 0.016). Adding SC and LC increased the digestibility of 7 and 2 AA by the distal half of the jejunum, respectively, and the digestibility of 8 and 13 AA by the distal half of the ileum, respectively (*P* ≤ 0.039). The β-diversity and abundance of some taxa were altered by addition of LC and SC in the ceca while no treatment effect on the ileal microbiota was found. The results give no indication of an incomplete release of the oil from the carriers because the TTR of most FA was increased upon addition of SC and LC. LC may be used to supply liposoluble feed additives without drawbacks for nutrient digestibility and growth while SC requires further examination.

## INTRODUCTION

Carrier materials are used to facilitate handling, dosing, and homogenous inclusion of liquid substances across various applications. In animal nutrition, carrier materials for oils in compound feeds may be used to supply feed additives such as fat-soluble vitamins. Inorganic substances, like silicon dioxide, are approved in the European Union (European Commission, 1999) and the United States (Food and Drug Administration, 2020) and can be used as carriers in animal feed.

While inorganic carriers are largely inert, organic carriers may contribute to the nutrient supply of animals. Potential alternatives to inorganic carriers are organic carriers based on lignocellulose, which are made from processed wood. Lignocellulose is used as water-insoluble fiber source in animal nutrition. As reviewed recently (Röhe and Zentek, 2021), lignocellulose can impact growth performance, nutrient digestibility, and the gut microbiota in poultry. We are aware of only one study investigating the influence of lignocellulose on the microbiota of broiler chickens using 16S ribosomal RNA (**rRNA**) sequencing (Zeitz et al., 2019). The authors reported minor changes in the cecal microbiota for one lignocellulose additive, while a decreased abundance of taxa belonging to Lachnospiraceae and Oscillospiraceae was observed for another lignocellulose additive, suggesting varying effects among lignocellulose additives (Zeitz et al., 2019).

A key consideration for using oil carriers in compound feeds is the complete release of the oil from the carrier in the digestive tract. However, we are not aware of any study investigating the influence of oil carriers in compound feeds in animal nutrition. Therefore, the objective of this study was to assess effects of oil carriers in compound feed on growth performance, energy utilization, fatty acid (**FA**) retention, amino acid (**AA**) digestibility, and gut microbiota in broiler chickens. We hypothesized that 1) both carriers decrease the total tract retention (**TTR**) of FA as an indicator for incomplete release of oil from the carriers and 2) LC influences the gut microbiota of the broiler chickens as a consequence of contained lignocellulose while effects of SC on the microbial composition are minor.

## MATERIAL AND METHODS

The experiment was conducted at the Agricultural Experiment Station of the University of Hohenheim in Eningen unter Achalm, Germany. All procedures were in accordance with the German animal welfare law and approved by the Regierungspräsidium Tübingen (approval number HOH66/21TE).

### Experimental Design

Six experimental diets were formulated for a 2 × 3 factorial arrangement of treatments. Rapeseed oil was included in diets at 20 g/kg or 40 g/kg. The rapeseed oil was either added without a carrier (**NC**), or in a mass ratio of 1:1 with an inorganic carrier based on silica (**SC**; Tixosil 38A, Solvay GmbH, Hannover, Germany) or an organic carrier based on lignocellulose (**LC**; J. Rettenmaier & Söhne GmbH + Co. KG, Rosenberg, Germany). Each experimental diet was tested in 8 replicates in a randomized complete block design, where blocks included possible effects of location within the barn. The design was optimized using the OPTEX procedure of SAS (version 9.4, SAS Institute Inc., Cary, NC).

### Experimental Diets

The experimental diets (Table 1) were based on corn, soybean meal, and wheat gluten meal and were calculated to meet or exceed the nutrient supply recommendations of the Gesellschaft für Ernährungsphysiologie (1999). Titanium dioxide was included as an indigestible marker. Cornstarch was added to the diets with 20 g/kg of rapeseed oil to achieve similar calculated concentrations of MEn in all experimental diets. Diamol was used to compensate for mass differences among the diets. The diets were mixed in the certified feed mill facilities at the Agricultural Experiment station of the University of Hohenheim. Diets were fed unpelleted aiming to avoid potential implications of the pelleting process on the oil-binding capacity of the carriers. The analyzed chemical compositions (Table 2) were similar to the formulated values.

**Table 1:** Composition of the experimental diets.

Oil inclusion	20 g/kg			40 g/kg		
Carrier^1^	NC	SC	LC	NC	SC	LC
*Feed composition (g/kg)*						
Corn	480.9	480.9	480.9	480.9	480.9	480.9
Soybean meal	350.0	350.0	350.0	350.0	350.0	350.0
Wheat gluten	50.0	50.0	50.0	50.0	50.0	50.0
ʟ-Lysine·sulfate	2.8	2.8	2.8	2.8	2.8	2.8
ᴅʟ-Methionine	2.2	2.2	2.2	2.2	2.2	2.2
ʟ-Threonine	0.6	0.6	0.6	0.6	0.6	0.6
ʟ-Arginine·HCl	0.3	0.3	0.3	0.3	0.3	0.3
ʟ-Valine	2.2	2.2	2.2	2.2	2.2	2.2
Monocalcium phosphate	11.2	11.2	11.2	11.2	11.2	11.2
Limestone (coarse)	4.4	4.4	4.4	4.4	4.4	4.4
Limestone (fine)	4.4	4.4	4.4	4.4	4.4	4.4
Vitamin premix^2^	2.0	2.0	2.0	2.0	2.0	2.0
Trace element premix^3^	0.5	0.5	0.5	0.5	0.5	0.5
Sodium bicarbonate	1.7	1.7	1.7	1.7	1.7	1.7
Sodium chloride	1.0	1.0	1.0	1.0	1.0	1.0
Choline chloride	0.8	0.8	0.8	0.8	0.8	0.8
Titanium dioxide	5.0	5.0	5.0	5.0	5.0	5.0
Rapeseed oil (refined)	20	-	-	40	-	-
SC/rapeseed oil mix^4^	-	40	-	-	80	-
LC/rapeseed oil mix^5^	-	-	40	-	-	80
Cornstarch	50	40	40	-	-	-
Diamol^6^	10	-	-	40	-	-
*Calculated composition (g/kg DM)*						
CP	262	261	260	260	261	260
Crude fat	51	51	51	71	71	71
Calcium	7.5	7.5	7.4	7.5	7.5	7.4
Total phosphorus	7.0	7.0	7.0	7.0	7.0	7.0

1 NC: no carrier; SC: silica-based carrier; LC: lignocellulose-based carrier.

2 Vitamin premix (Miavit GmbH, Essen, Germany) provided per kg of diet: 10,000 IU vitamin A as retinyl acetate; 3,000 IU vitamin D_3_ as cholecalciferol; 30 IU vitamin E as all rac-α-tocopherol; 2.4 mg vitamin K_3_ as menadione; 100 mg biotin; 1 mg folic acid; 3 mg thiamine; 6 mg riboflavin; 6 mg pyridoxine; 30 mg vitamin B12; 50 mg nicotinamide; 14 mg calcium d-pantothenate.

3 Trace element premix (Gelamin Gesellschaft für Tierernährung mbH, Memmingen, Germany) provided per kg of diet: 25 mg calcium from calcium carbonate; 80 mg manganese from manganese-(II)-oxide; 60 mg zinc from zinc sulphate; 25 mg iron from ferrous-(II)-sulphate monohydrate; 7.5 mg copper from cupric-(II)-sulphate pentahydrate; 0.6 mg iodine from calcium iodate; 0.2 mg selenium from sodium selenite; 15 mg sepiolite.

4 Rapeseed oil mixed with a silica-based carrier in a mass ratio of 1:1.

5 Rapeseed oil mixed with a lignocellulose-based carrier in a mass ratio of 1:1.

6 Purified diatomaceous earth mainly consisting of SiO_2_ (Imerys S.A., Paris, France).

**Table 2:** Analyzed chemical composition of experimental diets (g/kg DM, unless otherwise stated).

Oil inclusion	20 g/kg			40 g/kg		
Carrier^1^	NC	SC	LC	NC	SC	LC
DM (g/kg)	903	903	904	909	908	907
CP	263	263	269	263	269	263
Crude fat	54	54	55	75	76	75
Crude ash	70	81	60	99	102	62
Crude fiber	25	29	42	30	28	53
aNDFom^2^	98	101	112	97	92	124
ADFom^3^	24	28	39	28	20	54
ADL^4^	8	7	12	8	12	13
Gross energy (MJ/kg DM)	18.8	18.7	19.0	18.9	18.9	19.6
Calcium	7.7	7.8	7.4	7.7	7.7	7.5
Phosphorus	6.8	6.8	6.7	7.0	6.8	6.8
Titanium dioxide	5.6	5.4	5.2	5.9	5.3	5.2
Amino acids						
Alanine	12.5	12.4	12.6	12.3	12.4	12.6
Arginine	16.8	16.8	17.0	16.7	16.7	17.3
Aspartic acid + asparagine	25.7	25.8	26.3	25.7	25.8	26.6
Cysteine	3.8	3.9	3.8	4.0	3.8	4.4
Glutamic acid + glutamine	56.8	57.9	57.3	56.6	57.0	59.8
Glycine	11.0	11.0	11.1	10.9	10.9	11.2
Histidine	7.4	7.4	7.5	7.3	7.4	7.8
Isoleucine	11.7	11.6	11.9	11.6	11.7	11.8
Leucine	22.5	22.6	22.8	22.4	22.5	23.0
Lysine	15.1	15.0	15.4	15.3	15.0	15.3
Methionine	6.7	6.7	6.6	6.6	6.6	6.7
Phenylalanine	13.7	13.8	13.9	13.7	13.7	14.0
Proline	18.2	18.6	18.5	18.2	18.3	19.5
Serine	13.6	13.9	13.8	13.6	13.6	14.2
Threonine	10.6	10.7	10.7	10.6	10.6	10.9
Tyrosine	9.3	9.4	9.4	9.3	9.3	9.5
Valine	15.2	15.0	15.3	15.1	15.1	15.2
Fatty acids^5^ (mg/kg DM)						
C16:0	5,145	5,265	5,586	6,455	6,375	6,460
C16:1	79	80	84	128	131	129
C18:0	1,088	1,100	1,156	1,508	1,514	1,522
C18:1 c9	19,226	19,448	19,996	32,442	32,703	32,708
C18:2 c9,12	20,634	21,239	22,570	25,020	25,606	25,837
C18:3 c9,12,15	2,841	2,931	3,083	4,193	4,697	4,701
C20:0	227	229	233	352	357	357
C20:1 c11	342	338	358	591	621	616
C22:0	134	136	140	195	206	204

1 NC: no carrier; SC: silica-based carrier; LC: lignocellulose-based carrier.

2 Neutral detergent fiber, determined without residual ash and after treatment with α-amylase.

3 Acid detergent fiber, determined without residual ash.

4 Acid detergent lignin determined by solubilization of cellulose with sulfuric acid.

5 Values were below the limit of quantification of 60 mg/kg DM for C14:0, C17:0, C20:2 c11,14, C22:1 c13, C23:0, C24:1 and below the limit of detection of 20 mg/kg DM for C12:0, C15:0, C17:1, C18:1 t9, C18:2 t9,12, C20:3 c8,11,14, C20:4 c5,8,11,14, C24:0.

### Animals and Housing

Unsexed broiler hatchlings of the strain Ross 308 were obtained from a hatchery (Brüterei Süd ZN der BWE-Brüterei Weser-Ems GmbH & Co. KG, Regenstauf, Germany). The birds were housed in floor pens (3 × 4 m) on wood shavings and fed a conventional starter feed (Club Mastkükenstarter 4150020, Deutsche Tiernahrung Cremer GmbH & Co. KG, Mannheim, Germany; per kg 215 g CP, 10.5 g calcium, 5.5 g phosphorus, 12.5 MJ ME, 110 mg monensin sodium, 10 IU endo-1.4-β-xylanase [EC 3.2.1.8], and 750 FTU 6-phytase [EC 3.1.3.26]).

The experimental period started on d 18 of the experiment to ensure sufficient adaptation to the experimental diets until initiation of nutrient balancing on d 24. Fifteen animals each were distributed to 48 metabolism units (2 × 1 m) to achieve equal mean group weights and a similar weight distribution within each group. Feed and water were offered for ad libitum consumption throughout the experiment. The lighting regimen included 24 h lighting per day in the first 3 d and 18 h lighting per day thereafter. The temperature was set at 34°C for the first 3 d, reduced gradually to 19°C on d 21, and remained at this level until the end of the experiment.

### Experimental Procedures

The weight and feed consumption of the broiler chickens were recorded on d 18, 24, and 27. Spilled feed was collected daily, frozen at -20°C, dried at 103°C, and used to correct feed intake data. Total excreta were collected twice daily for 3 d from d 24 to 27, beginning at 7.00 h and 19.00 h. After removing contaminations, such as feathers and skin scales, the collected excreta were immediately frozen at -20°C.

On d 28, animals were anesthetized with a gas mixture (35% CO_2_, 35% N_2_, 30% O_2_) and euthanized with CO_2_. The digestive tract was immediately dissected and the jejunum, ileum, and ceca were removed. For digestibility determination, digesta from the distal half of the section from the end of the duodenum to Meckel's diverticulum (“jejunum”) and from the distal half of the section from Meckel's diverticulum to 2 cm anterior to the ileo-ceco-colonic junction (“ileum”) were rinsed out with ice-cold, double distilled water. Digesta were pooled from all birds of one unit, immediately frozen at -20°C, and later freeze dried. For microbiota analysis, both ceca and a 2 cm piece proximal to the section used to obtain ileal digesta for digestibility analysis were cut longitudinally. Digesta from these sections were obtained with a sterile spoon-spatula without scratching the mucosa. Digesta were pooled from all birds of one unit, homogenized, and stored at -80°C.

### Chemical and Microbiota Analysis

Feed, digesta, and excreta samples were ground with a ball mill (MM400, Retsch GmbH, Haan, Germany) for analysis of AA, FA, energy, and titanium. A centrifugal mill (ZM200, Retsch GmbH) equipped with a 0.5 mm screen was used for all other analyses. Official analysis methods in Germany (Verband Deutscher Landwirtschaftlicher Untersuchungs- und Forschungsanstalten, 2007) were used to analyze DM (no. 3.1), CP (no. 4.1.1), crude fat (no. 5.1.1), crude ash (no. 8.1), crude fiber (no. 6.1.1), and detergent fiber fractions (no. 6.5). Methods established in our lab were applied for the analysis of calcium, phosphorus, titanium (Zeller et al., 2015), AA (Siegert et al., 2017), and FA (Hoffmann et al., 2013). Gross energy (**GE**) was analyzed using a calorimeter (IKA C200, IKA-Werke GmbH & CO. KG, Staufen, Germany) and benzoic acid as the standard.

For microbiota analysis, the DNA of the digesta samples was extracted using the FastDNA SPIN Kit for Soil (MP Biomedicals LLC, Solon, OH) according to the manufacturer's instructions with some slight modifications as described by Burbach et al. (2016) and as follows. Sampled digesta were initially homogenized with a FastPrep-24 5G Instrument (MP Biomedicals LLC, Solon, OH) twice for 40 s at 6.0 m/s. For DNA binding after protein precipitation, SPIN filters were centrifugated at 14,000 × *g* for 5 min after each time of adding the solution. The quantity and quality of the extracted DNA were measured with a NanoDrop One (Thermo Fisher Scientific, Wilmington, DE).

The amplification of the V1-2 region of the 16S rRNA gene and Illumina library preparation was performed with a 2-step PCR according to the protocol described by Kaewtapee et al. (2017). The PCR amplicons were checked with agarose gel electrophoresis, normalized with SequalPrep Normalization Plate Kit (Invitrogen Corporation, Carlsbad, CA), and purified with MinElute PCR Purification Kit (Qiagen N.V., Hilden, Germany). The libraries were sequenced using an Illumina MiSeq PE250 platform (Illumina Inc., San Diego, CA).

The raw sequencing data were demultiplexed, trimmed, denoised, and taxonomically assigned as described recently (Sáenz et al. 2023) using QIIME2 (version 2022.2; Bolyen et al., 2019) and the SILVA SSU reference database (release 138.1 Ref NR 99; Quast et al., 2013; Yilmaz et al., 2014). Significant amplicon sequence variants (**ASV**) were annotated using NCBI BLAST and the 16S rRNA database for bacteria (Johnson et al., 2008).

### Calculations and Statistical Analysis

The digestibility of nutrients by the distal half of the jejunum and the distal half of the ileum was calculated using the following equation:

(1) $$\text{Digestibility}=100-\left[\left(Ti_{\text{diet}}\times \text{nutrien}t_{\text{digesta}}\right)/\left(Ti_{\text{digesta}}\times \text{nutrien}t_{\text{diet}}\right)\right]\times 100$$

where, nutrient_diet_ and nutrient_digesta_were the nutrient concentrations in the diet and digesta, respectively, and Ti_diet_ and Ti_digesta_ were the titanium dioxide concentrations in the diet and digesta, respectively. The TTR of nutrients and MEn were calculated with the following equations:

(2) TTR=[intake(g/d)−excretion(g/d)]/intake(g/d)

(3) $$\begin{array}{l} \text{MEn}\;\left(\text{MJ}/\text{kg DM}\right)=\\ \left\{\text{energy}\;\text{intake}\;\left(\text{MJ}/d\right)-\text{energy}\;\text{excretion}\;\left(\text{MJ}/d\right)-36.5\;\text{MJ}/\text{kg}\;N\times \left[N\;\text{intake}\;\left(\text{kg}/d\right)-N\;\text{excretion}\;\left(\text{kg}/d\right)\right]\right\}\\ /\;\text{feed}\;\text{intake}\;\left(kg\;DM/d\right) \end{array}$$

Data were statistically evaluated using the MIXED procedure in SAS with the metabolism units as the statistical unit and the following model:

(4) $$y_{ijk}=\mu +\alpha_{i}+\beta_{j}+\alpha \beta_{ij}+b_{k}+e_{ijk}$$

where, *y_ijk_* is the response trait; *µ* is the mean; *α_i_* is the fixed carrier effect (*i* = NC, SC, or LC); *β_j_* is the fixed effect of the oil inclusion (*j* = 20 g/kg or 40 g/kg); *b_k_* is the random effect of block *k*, which was included if the consideration of the block effect reduced the Akaike information criterion; and *e_ijk_* is the residual error.

The α-diversity metrics and β-diversity distance matrices were calculated in QIIME2 with a sampling depth of 32,000. One cecal sample of the treatment SC with 20 g/kg oil inclusion was excluded due to a lower number of reads. The statistical analysis for the microbiota data was performed in R (version 4.2.2; R Core Team, 2022). For statistical comparison of α-diversity metrics, an ANOVA was performed with the “car” (Fox and Weisberg, 2019) or “lmerTest” (Kuznetsova et al., 2017) package. Permutational multivariate analysis of variance (**PERMANOVA**) was performed using the “adonis2” function of the “vegan” package (Oksanen et al., 2022) for analysis of β-diversity. The block effect was considered by restricting permutations of PERMANOVA within blocks using the “strata” argument. Nonmetric multidimensional scaling (**NMDS**) was used to visualize the β-diversity with the “metaMDS” function of the “vegan” package.

The “ALDEx2” package (Fernandes et al., 2014) was chosen due to its high consistency across different datasets and low false discovery rate in a recent comparison (Nearing et al., 2022) for differential abundance analysis. The “aldex.glm” function was used for treatment comparisons. *P*-values were corrected with the Holm-Bonferroni method. Statistical significance was set at *P* < 0.050 for all response traits.

## RESULTS

### Animal Growth Performance and Energy

The oil inclusion × carrier interaction was significant for ADG, ADFI, and gain:feed ratio (**G:F**, *P* ≤ 0.003; Table 3). Addition of SC decreased ADG, ADFI, and G:F compared to NC and LC at 20 g/kg and 40 g/kg oil inclusion (*P <* 0.001). Increasing the oil inclusion increased ADG for NC and LC (*P* ≤ 0.006). For LC, 40 g/kg oil inclusion raised ADG and G:F compared to 20 g/kg (*P* ≤ 0.025). The higher oil inclusion reduced ADFI for SC (*P* = 0.048). There was a significant interaction for MEn (*P* = 0.005) but not for the GE digestibility by the distal half of the jejunum or the distal half of the ileum (Table 3). The MEn was higher for LC compared to NC and SC at 40 g/kg oil inclusion (*P* ≤ 0.036). No treatment differences for MEn were observed at 20 g/kg oil inclusion. For LC, the MEn was increased at 40 g/kg compared to 20 g/kg oil inclusion (*P* < 0.001). The digestibility of GE by the distal half of the jejunum and the distal half of the ileum was increased for SC and decreased for LC (*P* ≤ 0.026). There was no effect of the oil inclusion on GE digestibility.

**Table 3:** Influence of the oil inclusion and carrier on ADG, ADFI, gain:feed ratio (**G:F**), energy (**GE**) digestibility by the distal half of the jejunum and the distal half of the ileum, and MEn in broiler chickens.

Oil inclusion	Carrier^1^	ADG (g/d)	ADFI (g/d)	G:F (g/g)	GE digestibility jejunum (%)	GE digestibility ileum (%)	MEn (MJ/kg DM)
Treatments (n = 8)							
20 g/kg	NC	67.4^bc^	99.5ᵇ	0.677^b^	58	72	15.6bcd
	SC	59.0^d^	90.7^c^	0.650^c^	61	73	15.6^cd^
	LC	66.8^c^	98.0^b^	0.682^b^	58	71	15.5^d^
40 g/kg	NC	71.7^a^	105.0^a^	0.682^b^	62	77	15.7^bc^
	SC	56.1^d^	87.3^d^	0.642^c^	62	73	15.8^b^
	LC	70.2^ab^	99.3^b^	0.707^a^	57	70	16.0^a^
SEM		1.3	1.2	0.007	1.0	0.5	0.050
Main effects^2^							
20 g/kg		.	.	.	59	72	.
40 g/kg		.	.	.	60	72	.
SEM					0.8	0.4	
	NC	.	.	.	60^b^	72^b^	.
	SC	.	.	.	62^a^	73^a^	.
	LC	.	.	.	57^c^	70^c^	.
	SEM				0.8	0.4	
*P*-Values							
Oil inclusion		0.063	0.248	0.044	0.130	0.523	<0.001
Carrier		<0.001	<0.001	<0.001	<0.001	<0.001	0.539
Oil inclusion × carrier		0.002	0.002	0.003	0.550	0.235	0.005

a-d Values in the same column with different superscripts are significantly different (*P* < 0.050).

1 NC: no carrier; SC: silica-based carrier; LC: lignocellulose-based carrier.

2 Presented if the oil inclusion × carrier interaction was not significant (*P* > 0.050); n = 24 for oil inclusion; n = 16 for carrier.

### Total Tract Retention of Crude Fat and Fatty Acids

The oil inclusion × carrier interaction was significant for the TTR of crude fat (*P* = 0.022; Table 4). The TTR of crude fat was higher for SC than for NC and LC at 20 g/kg and 40 g/kg oil inclusion (*P* ≤ 0.019). Increasing the oil inclusion raised the TTR of crude fat for NC, SC, and LC (*P* < 0.001). The interactions for the TTR of the FA C16:0, C18:1, C18:3, C20:0, and C20:1 were significant (*P* ≤ 0.046), but not for C18:0, C18:2, and C22:0. Addition of SC and LC increased the TTR of C18:2 compared to NC (*P* ≤ 0.016). The addition of SC also increased the TTR of C22:0 (*P* = 0.002). Increasing the oil inclusion raised the TTR of the FA C18:0, C18:2, and C22:0 (*P* < 0.001). At 20 g/kg and 40 g/kg oil inclusion, addition of SC and LC increased the TTR of C18:1 (*P* ≤ 0.024), and addition of SC increased the TTR of C18:3 and C20:1 compared to NC (*P* ≤ 0.024). At an oil inclusion of 20 g/kg, the TTR of C18:3 was increased by addition of LC compared to NC (*P* = 0.004). For NC, SC, and LC, the higher oil inclusion increased the TTR of C18:1, C18:3, C20:0, and C20:1 (*P* ≤ 0.033) but decreased the TTR of C16:0 (*P* < 0.001).

**Table 4:** Influence of the oil inclusion and the carrier on the total tract retention of crude fat and fatty acids detected above the limit of quantification in the excreta of broiler chickens.

Oil inclusion	Carrier^1^	Crude fat	C16:0	C18:0	C18:1 c9	C18:2c9,12	C18:3 c9,12,15	C20:0	C20:1 c11	C22:0
Treatments (n = 8)										
20 g/kg	NC	79^d^	91^a^	68	83^e^	73	89^d^	70^c^	86^d^	75
	SC	81^c^	84^b^	69	85^d^	75	90^c^	71^c^	87^bc^	77
	LC	79^d^	83^b^	69	85^d^	75	90^c^	71^c^	87^cd^	75
40 g/kg	NC	83^b^	72^c^	74	87^c^	77	90^bc^	77^b^	87^bc^	79
	SC	86^a^	68^d^	76	91^a^	79	93^a^	80^a^	91^a^	83
	LC	83^b^	72^c^	74	88^b^	78	91^b^	77^b^	88^b^	79
SEM		0.4	1.1	0.7	0.3	0.5	0.3	0.6	0.5	0.7
Main effects^2^										
20 g/kg		.	.	69^b^	.	74^b^	.	.	.	76^b^
40 g/kg		.	.	75^a^	.	78^a^	.	.	.	80^a^
SEM				0.7		0.3				0.4
	NC	.	.	71	.	75^b^	.	.	.	77^b^
	SC	.	.	73	.	77^a^	.	.	.	80^a^
	LC	.	.	72	.	76^a^	.	.	.	77^b^
	SEM			0.5		0.4				0.5
*P*-values										
Oil inclusion		<0.001	<0.001	0.072	<0.001	0.013	<0.001	<0.001	<0.001	<0.001
Carrier		<0.001	<0.001	<0.001	<0.001	<0.001	<0.001	<0.001	<0.001	<0.001
Oil inclusion × carrier		0.022	0.004	0.212	0.015	0.393	0.005	0.046	0.006	0.209

a-e Values in the same column with different superscripts are significantly different (*P*<0.050).

1 NC: no carrier; SC: silica-based carrier; LC: lignocellulose-based carrier.

2 Presented if the oil inclusion × carrier interaction was not significant (*P* > 0.050); n = 24 for oil inclusion; n = 16 for carrier.

### Crude Protein and Amino Acid Digestibility

The interaction effects for the digestibility of all AA were not significant, except for the digestibility of Cys by the distal half of the jejunum (*P* = 0.004) and Arg, Cys, His, and Pro by the distal half of the ileum (*P* ≤ 0.038; Tables 5 and 6). Adding SC compared to NC increased the digestibility of CP and 7 AA by the distal half of the jejunum (Arg, Gln+Glu, His, Leu, Met, Ser, and Pro; *P* ≤ 0.039) and the digestibility of CP and 8 AA by the distal half of the ileum (Gln+Glu, Gly, Ile, Leu, Lys, Met, Phe, and Ser; *P* ≤ 0.032). Addition of LC increased the digestibility of Pro and His by the distal half of the jejunum (*P* ≤ 0.011) and the digestibility of CP and 13 AA by the distal half of the ileum (Ala, Asn+Asp, Gln+Glu, Gly, Ile, Leu, Lys, Met, Phe, Ser, Thr, Tyr, and Val; *P* ≤ 0.007). Increasing the oil inclusion raised the digestibility of Pro by the distal half of the jejunum (*P* = 0.045) and the digestibility of CP and 6 AA by the distal half of the ileum (Asn+Asp, Ile, Leu, Phe, Ser, and Thr; *P* ≤ 0.043).

**Table 5:** Influence of the oil inclusion and the carrier on the digestibility of essential amino acids by the distal half of the jejunum (J) and the distal half of the ileum (I).

Oil inclusion	Carrier^1^	Lys		Met		Thr		Val		Ile		Leu		His		Arg		Phe	
		J	I	J	I	J	I	J	I	J	I	J	I	J	I	J	I	J	I
Treatments (n = 8)																			
20 g/kg	NC	73	86	83	92	61	76	72	85	69	84	70	85	66	81^c^	75	87^c^	70	85
	SC	76	87	84	92	64	77	73	85	71	84	72	85	69	81^c^	77	88^bc^	73	86
	LC	75	87	84	92	62	77	73	86	71	85	72	86	68	84^b^	77	89^ab^	72	86
40 g/kg	NC	75	86	83	91	63	77	73	85	70	84	71	85	66	80^d^	75	87^d^	72	86
	SC	75	87	84	92	63	77	73	86	71	85	72	86	68	82^c^	77	88^ab^	72	87
	LC	74	88	83	92	62	78	72	86	69	86	71	87	69	85^a^	76	89^a^	72	87
SEM		0.7	0.4	0.4	0.3	0.9	0.7	0.6	0.5	0.7	0.5	0.7	0.4	0.9	0.6	0.6	0.4	0.7	0.4
Main effects^2^																			
20 g/kg		75	87	84	92	62	77^b^	73	85	70	84^b^	72	85^b^	68	.	76	.	72	86^b^
40 g/kg		75	87	83	92	63	78ᵃ	73	86	70	85^a^	72	86^a^	68	.	76	.	72	87^a^
SEM		0.5	0.3	0.3	0.2	0.6	0.6	0.4	0.4	0.5	0.4	0.5	0.3	0.7		0.5		0.5	0.3
	NC	74	86^b^	83^b^	91^b^	62	76^b^	73	85^b^	70	84^b^	71^b^	85^c^^3^	66^b^	.	75^b^	.	71	86^b^^4^
	SC	75	87^a^	84^a^	92^a^	63	77ᵃb	73	85ᵃb	71	85^a^	72^a^	86^b^^3^	69^a^	.	77^a^	.	72	86^a^^4^
	LC	74	88^a^	83b	92^a^	62	78^a^	73	86^a^	70	85^a^	72^ab^	86^a^^3^	69^a^	.	76^ab^	.	72	87^a^^4^
	SEM	0.6	0.3	0.4	0.3	0.7	0.6	0.5	0.4	0.6	0.4	0.5	0.4	0.8		0.5		0.6	0.3
*P*-Values																			
Oil inclusion		0.733	0.091	0.130	0.701	0.514	0.011	0.773	0.071	0.958	0.019	0.932	0.018	0.878	0.860	0.449	0.820	0.636	0.007
Carrier		0.059	<0.001	0.010	0.002	0.115	0.024	0.406	0.007	0.233	0.002	0.037	<0.001	<0.001	<0.001	0.011	<0.001	0.052	0.001
Oil inclusion × carrier		0.090	0.489	0.343	0.125	0.130	0.710	0.071	0.458	0.257	0.368	0.562	0.189	0.775	0.001	0.563	0.038	0.301	0.197

a-d Values in the same column with different superscripts are significantly different (*P* < 0.05).

1 NC: no carrier; SC: silica-based carrier; LC: lignocellulose-based carrier.

2 Presented if the oil inclusion × carrier interaction was not significant (*P* > 0.050); n = 24 for oil inclusion; n = 16 for carrier.

3 85.0^c^ for NC, 85.7^b^ for SC, and 86.5^a^ for LC.

4 85.5^b^ for NC, 86.3^a^ for SC, and 86.8^a^ for LC.

**Table 6:** Influence of the oil inclusion and the carrier on the digestibility of CP and nonessential amino acids by the distal half of the jejunum (J) and the distal half of the ileum (I).

Oil inclusion	Carrier^1^	CP		Ala		Asn+Asp		Cys		Gln+Glu		Gly		Pro		Ser		Tyr	
		J	I	J	I	J	I	J	I	J	I	J	I	J	I	J	I	J	I
Treatments (n = 8)																			
20 g/kg	NC	67	82	67	83	60	77	45^c^	65^c^	76	88	58	76	67	84^c^	64	80	68	83
	SC	70	82	69	83	64	78	49^b^	67^b^	78	89	62	77	70	84^c^	67	81	71	84
	LC	68	83	68	84	63	79	46^bc^	67^b^	77	89	60	78	69	86^b^	66	81	70	84
40 g/kg	NC	69	82	68	83	63	78	48^b^	65^c^	77	88	60	76	70	84^c^	66	80	70	84
	SC	70	84	69	84	64	79	47^bc^	67^bc^	78	89	61	77	70	84^c^	67	81	70	84
	LC	68	83	68	84	63	80	53^a^	71^a^	77	90	60	79	72	87^a^	66	82	69	85
SEM		0.8	0.5	0.8	0.5	0.1	0.7	1.6	0.1	0.7	0.4	1.1	0.8	0.9	0.5	0.8	0.6	0.7	0.5
Main effects^2^																			
20 g/kg		69	82^b^	68	83	62	78^b^	.	.	77	89	60	77	69^b^	.	66	80^b^	70	84
40 g/kg		69	83^a^	68	84	63	79^a^	.	.	77	89	61	77	70^a^	.	66	81^a^	70	84
SEM		0.6	0.4	0.5	0.5	0.7	0.6			0.5	0.3	0.8	0.7	0.7		0.6	0.5	0.5	0.4
	NC	68^b^	82^b^	68	83^b^	62	77^b^	.	.	76^b^	88^b^	59	76^c^	68^b^	.	65^b^	80^b^	69	83^b^
	SC	70^a^	83^a^	69	83^ab^	64	78^b^	.	.	78^a^	89^a^	62	77^b^	70^a^	.	67^a^	81^a^	70	84^ab^
	LC	68^b^	83^a^	68	84^a^	63	79^a^	.	.	77^ab^	90^a^	60	78^a^	70^a^	.	66^ab^	82^a^	69	85^a^
	SEM	0.7	0.4	0.6	0.5	0.8	0.6			0.6	0.4	0.8	0.7	0.7		0.7	0.6	0.6	0.4
*P*-Values																			
Oil inclusion		0.588	0.023	0.762	0.057	0.299	0.043	0.007	0.174	0.330	0.100	0.526	0.142	0.045	0.397	0.416	0.021	0.916	0.039
Carrier		0.033	<0.001	0.065	0.004	0.059	<0.001	0.058	<0.001	0.028	<0.001	0.059	<0.001	0.027	<0.001	0.013	<0.001	0.171	0.007
Oil inclusion × carrier		0.176	0.192	0.489	0.279	0.155	0.967	0.004	0.004	0.244	0.377	0.344	0.590	0.122	0.013	0.135	0.311	0.197	0.403

a-c Values in the same column with different superscripts are significantly different (*P* < 0.050).

1 NC: no carrier; SC: silica-based carrier; LC: lignocellulose-based carrier.

2 Presented if the oil inclusion × carrier interaction was not significant (*P* > 0.050); n = 24 for oil inclusion; n = 16 for carrier.

### Effects on the Microbiota

A total of 5,688,015 forward and backward reads were obtained following the demultiplexing step. After denoising, 4,460 ASVs were identified, which were reduced to 1,657 after filtering spurious ASVs. Of all ASVs, 98.8% could be assigned to an order, 97.3% to a family, 77.0% to a genus, and 65.1% to a species.

There was no significant interaction for α-diversity metrics in the ileal or cecal digesta (Table 7). The number of observed ASVs in the ceca was influenced by the carrier (*P* = 0.031). The number of observed ASVs was higher for SC than for NC (*P* = 0.024). Faith's phylogenetic diversity (**PD**) was significantly affected by the carrier and the oil inclusion (*P* = 0.021). Faith's PD for SC was higher than for NC (*P* = 0.017). At an oil inclusion of 20 g/kg, Faith's PD was higher than at 40 g/kg (*P* = 0.021). No significant treatment effects were detected in the ileum.

**Table 7:** Summary statistics and results of statistical comparisons of α-diversity metrics of ileal and cecal digesta samples.

Oil inclusion	Carrier^1^	Ileum			Ceca		
		Observed ASVs^2^	Faith's PD^3^	Shannon	Observed ASVs^2^	Faith's PD^3^	Shannon
Treatments (n = 8)							
20 g/kg	NC	90.8	10.2	4.06	627	39.2	6.96
	SC	79.8	8.63	4.07	669	39.9	7.12
	LC	80.0	8.84	4.05	644	40.0	7.11
40 g/kg	NC	87.2	9.23	4.28	573	37.1	6.68
	SC	87.8	8.60	4.25	655	40.4	7.18
	LC	84.1	9.28	4.22	613	37.5	7.05
SEM		5.0	0.88	0.16	29	0.9	0.14
Main effects^4^							
20 g/kg		83.5	9.23	4.06	643	39.7^a^	7.06
40 g/kg		86.5	9.04	4.25	614	38.3^b^	6.97
SEM		2.9	0.51	0.09	17	0.5	0.08
	NC	89.0	9.72	4.17	600^b^	38.1^b^	6.82
	SC	83.3	8.61	4.16	656^a^	40.1^a^	7.15
	LC	81.9	9.06	4.14	629^ab^	38.8^ab^	7.08
	SEM	3.6	0.62	0.11	20	0.7	0.1
*P*-values							
Oil inclusion		0.303	0.710	0.271	0.088	0.021	0.507
Carrier		0.155	0.219	0.994	0.031	0.021	0.093
Oil inclusion × carrier		0.257	0.514	0.983	0.458	0.053	0.352

a,b Values in the same column with different superscripts are significantly different (*P* < 0.050).

1 NC: no carrier; SC: silica-based carrier; LC: lignocellulose-based carrier.

2 ASV: amplicon sequence variant.

3 PD: phylogenetic diversity.

4 n = 24 for oil inclusion; n = 16 for carrier.

**Figure 1:**
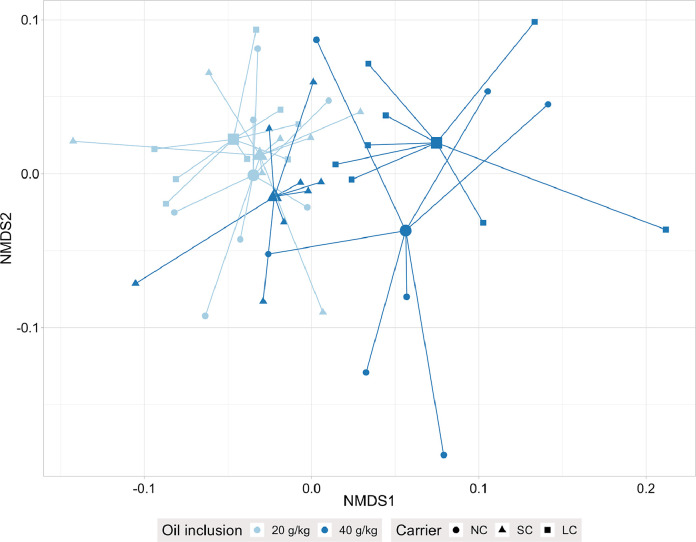
Nonmetric multidimensional scaling of unweighted UniFrac of cecal digesta samples taken from broiler chickens fed 20 g/kg or 40 g/kg rapeseed oil combined with no carrier (**NC**), a silica-based carrier (SC), or a lignocellulose-based carrier (**LC**), analyzed by 16S ribosomal RNA sequencing. The symbols bigger in size represent centroids, stress value=0.2271 (n=8 per treatment).

The β-diversity analysis showed a significant interaction for the unweighted and the weighted UniFrac (*P* = 0.002) but not for the Bray-Curtis dissimilarity in the ceca (Table 8). The oil inclusion and the carrier affected Bray-Curtis dissimilarity (*P* = 0.001). Pairwise comparisons of the carriers showed significant differences among NC, SC, and LC (*P* ≤ 0.008). NC and LC at 40 g/kg oil inclusion clustered separately from the other treatments in NMDS of unweighted UniFrac (Figure 1). Pairwise comparisons by PERMANOVA showed a significant difference between NC and LC at an oil inclusion of 40 g/kg (*P* ≤ 0.039) and between NC at 20 g/kg and 40 g/kg oil inclusion (*P* ≤ 0.039) for unweighted and weighted UniFrac (Table 8). The β-diversity in the ileum was not significantly affected by treatment.

**Table 8:** *P*-Values and R² for main effects and pairwise comparisons of carriers and treatments by PERMANOVA for different β-diversity metrics in ileum and ceca.

	Ileum						Ceca					
	Bray-Curtis		Unweighted UniFrac		Weighted UniFrac		Bray-Curtis		Unweighted UniFrac		Weighted UniFrac	
	*P*	R^2^	*P*	R^2^	*P*	R^2^	*P*	R^2^	*P*	R^2^	*P*	R^2^
Main effects^1^												
Oil inclusion	0.053	0.029	0.415	0.020	0.249	0.017	0.001	0.064	0.001	0.053	0.001	0.053
Carrier	0.196	0.041	0.337	0.043	0.251	0.032	0.001	0.085	0.001	0.081	0.001	0.081
Oil inclusion × carrier	0.774	0.023	0.675	0.034	0.500	0.025	0.250	0.041	0.002	0.060	0.002	0.060
Carrier (n=16)^2^												
NC - SC	.	.	.	.	.	.	0.008	0.059	.	.	.	.
SC - LC	.	.	.	.	.	.	0.003	0.062	.	.	.	.
LC - NC	.	.	.	.	.	.	0.003	0.073	.	.	.	.
Treatments (n = 8)^3^												
NC + 20 g/kg vs. SC + 20 g/kg	.	.	.	.	.	.	.	.	0.047	0.085	0.414	0.065
NC + 20 g/kg vs. LC + 20 g/kg	.	.	.	.	.	.	.	.	0.020	0.089	0.414	0.060
NC + 20 g/kg vs. NC + 40 g/kg	.	.	.	.	.	.	.	.	0.013	0.145	0.039	0.145
NC + 40 g/kg vs. SC + 40 g/kg	.	.	.	.	.	.	.	.	0.013	0.125	0.078	0.194
NC + 40 g/kg vs. LC + 40 g/kg	.	.	.	.	.	.	.	.	0.013	0.148	0.039	0.244

1 n = 24 for oil inclusion; n = 16 for carrier.

2 Pairwise comparisons of carriers, presented if the oil inclusion × carrier interaction was not significant and carrier effect was significant; NC, no carrier; SC, silica-based carrier; LC, lignocellulose-based carrier.

3 Pairwise comparisons of treatments, presented if the oil inclusion × carrier interaction was significant (*P* < 0.050).

**Figure 2:**
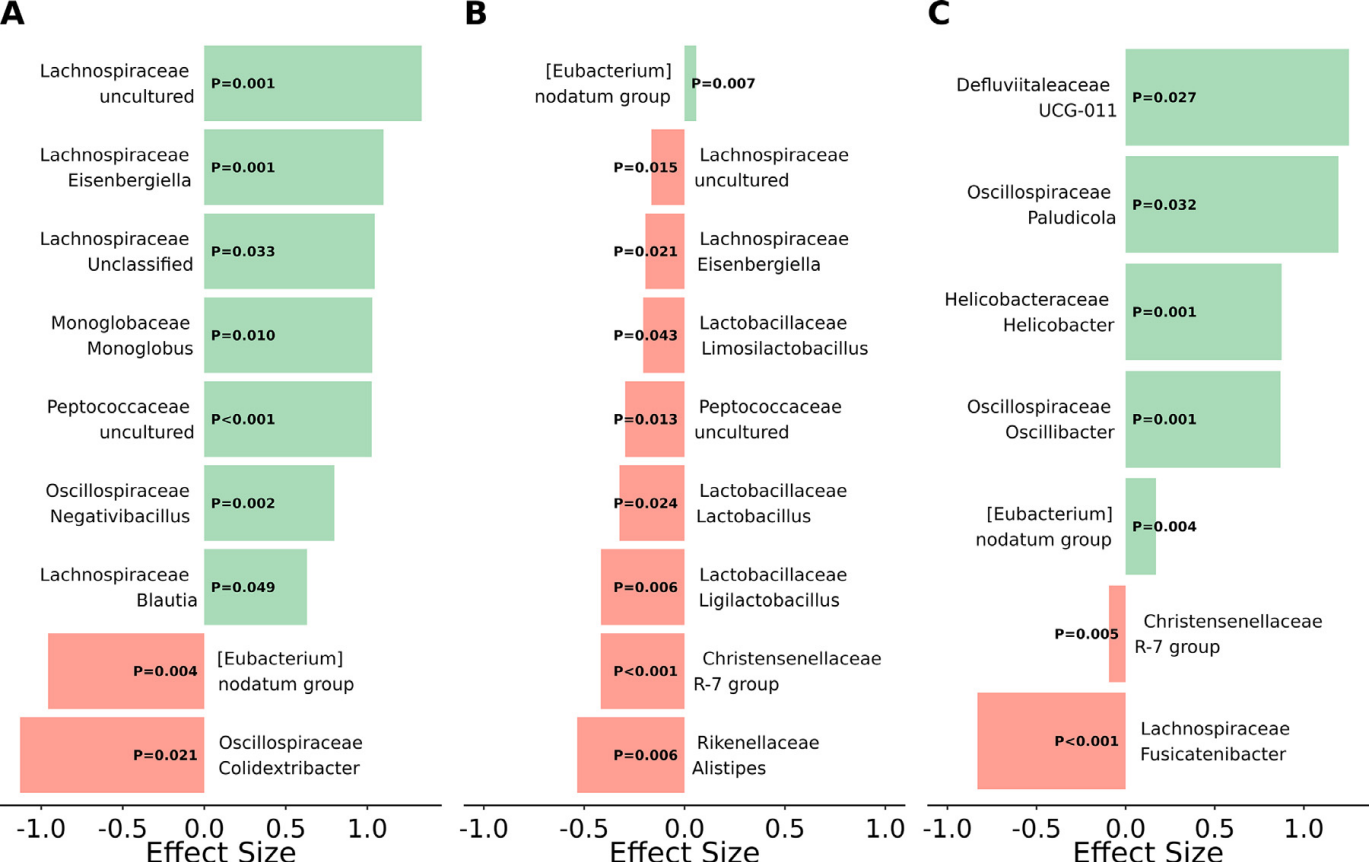
Effect sizes of significantly differential abundant genera detected in cecal samples taken from broiler chickens fed 20 g/kg or 40 g/kg of rapeseed oil combined with no carrier (**NC**), a silica-based carrier (SC), or a lignocellulose-based carrier (**LC**), analyzed by 16S ribosomal RNA sequencing. Treatments were compared pairwise by ALDEx2 (n = 8 per treatment). (A) Comparison of NC at 20 g/kg oil inclusion to NC at 40 g/kg oil inclusion. (B) Comparison of NC at 40 g/kg oil inclusion to SC at 40 g/kg oil inclusion. (C) Comparison of NC at 40 g/kg oil inclusion to LC at 40 g/kg oil inclusion.

Differential abundance analysis showed significant differences between treatment comparisons in the ceca (Supplemental file), but no effects in the ileum. Most taxa were differentially abundant between NC and LC at 40 g/kg oil inclusion. Some taxa of Firmicutes, including Defluviitaleaceae UCG-011 (0.3 vs. 0.1%), *Oscillibacter* (0.5 vs. 0.2%), and *Paludicola* (0.4 vs. 0.1%) were increased for LC compared to NC at 40 g/kg oil inclusion (*P* ≤ 0.032), while *Fusicatenibacter* (0.3 vs. 0.8%) and *Clostridium spiroforme* (0.2 vs. 0.4%) were decreased (*P* ≤ 0.008; Figure 2). An ASV identified as *Helicobacter canadensis* with 99.66% identity by BLAST alignment was increased by addition of LC compared to NC at 20 g/kg (0.7 vs. 0.2%) and 40 g/kg oil inclusion (0.7 vs. 0.1%; *P* ≤ 0.025). The families Christensenellaceae (0.6 vs. 1.1%), Rikenellaceae (20.9 vs. 27.0%), and Lactobacillaceae (8.0 vs. 12.2%) as well as their genera *Ligilactobacillus* (3.9 vs. 5.8%), *Lactobacillus* (3.7 vs. 5.3%), *Limosilactobacillus* (0.4 vs. 1.1%), and *Alistipes* (19.3 vs. 24.8%) were decreased by addition of SC at 40 g/kg oil inclusion (*P* ≤ 0.043). For NC, 40 g/kg compared to 20 g/kg oil inclusion increased the taxa *Monoglobus* (0.4 vs. 0.2%), *Blautia* (2.3 vs. 1.7%), *Eisenbergiella* (0.7 vs. 0.3%), *Negativibacillus* (1.3 vs. 0.8%), uncultured Peptococcaceae (0.2 vs. 0.1%), uncultured Lachnospiraceae (4.0 vs. 1.8%), and unclassified Lachnospiraceae (12.5 vs. 9.8%; *P* ≤ 0.049), and decreased *Colidextribacter* (0.3 vs. 0.7%; *P* ≤ 0.021).

## DISCUSSION

Results of the present study give no indication of an incomplete release of the oil from the carriers, since both carriers increased or had no effect on the TTR of crude fat and all FA, except for C16:0. Therefore, the first hypothesis was rejected. Nonetheless, it cannot be ruled out that an incomplete release of the oil from the carriers was not detected. Such a phenomenon would have materialized if the increase in disappearance of the released oil upon the presence of the carriers was bigger than the constraint by an incomplete oil release. Adding LC and SC altered the cecal microbiota while no changes in the ileal microbiota were detected. A more pronounced effect upon addition of LC than of SC is suggested by larger differences in β-diversity and higher effect sizes of differentially abundant taxa in the ceca. Hence, the second hypothesis was confirmed.

### Effects of the Lignocellulose-Based Carrier

The increase in TTR of FA and AA digestibility upon addition of LC may result from the contained lignocellulose and the large particle size of LC in consequence of increased enzymatic digestion. As reviewed by Röhe and Zentek (2021), results on effects of lignocellulose supplementation to poultry diets are conflicting in the literature. A study described increased crude fat digestibility by the ileum while other studies reported unaffected or reduced nutrient and energy digestibility upon lignocellulose supplementation. However, the results of preliminary studies on lignocellulose might not be comparable to the present study, since lignocellulose additives are usually fed as fine particles (Röhe and Zentek, 2021), whereas LC in this study represented coarse particles. As reviewed by Rodrigues and Choct (2018), addition of insoluble fiber and increased particle size of the feed can increase the retention time of the feed in the proventriculus and gizzard and intensify gastroduodenal reflux. Consequences of more pronounced gastroduodenal reflux include a longer exposure time of the digesta to enzymes, resulting in higher nutrient digestibility. Prolonged retention time in the proventriculus and gizzard were described to lead to increased secretion of hydrochloric acid and proventricular enzymes (Rodrigues and Choct, 2018). Further, elevated activities of pancreatic amylase (Hetland et al., 2003) and proteolytic enzymes (Yokhana et al., 2016) were reported upon insoluble fiber supplementation in poultry.

The LC may have increased microbial fermentation in the ceca and thus contributed to the energy supply of the host through production of short-chain fatty acids (**SCFA**). We observed an increased abundance of members of the family Oscillospiraceae in the ceca of broiler chickens at 40 g/kg oil inclusion when LC was added. Oscillospiraceae are associated with the production of butyrate (Medvecky et al., 2018), which is a relevant energy source of colonocytes (reviewed by Maki et al., 2019). An increased abundance of butyrate-producing bacteria in the cecal microbiota upon lignocellulose supplementation was reported for laying hens (Hou et al., 2020; Sun et al., 2021, 2022), whereas Zeitz et al. (2019) detected an enrichment of lactate-producing bacteria in broiler chickens. The changes in the cecal microbiota observed in the present study and in the literature indicate that the lignocellulose in LC may act as substrate for microbial fermentation. In the present study, increased MEn was determined for LC compared to NC at 40 g/kg oil inclusion, which cannot be solely explained by the additional energy provided by the higher digestibility of AA and FA. Further, the increased MEn determined for LC compared to NC at 40 g/kg oil inclusion was not caused by GE digestibility by the distal half of the ileum because GE digestibility by the distal half of the ileum was lowest for LC. This suggests that increased MEn originated from postileal processes. Therefore, the changes in the cecal microbiota may have contributed to the energy supply of the broiler chickens. However, it needs to be elucidated if LC acted as a substrate for the cecal microbiota since only small or soluble particles can enter the ceca (Svihus et al., 2013). It remains open why there was no effect on MEn for LC at 20 g/kg oil inclusion. Possibly, higher grinding activity of the gizzard for LC at 40 g/kg than at 20 g/kg oil inclusion led to more small particles that could enter the ceca.

The only taxon being affected by addition of LC at both oil levels was *H. canadensis*, but the relevance of this observation is unclear. *H. canadensis* was suggested as a zoonotic pathogen since it is closely related to pathogenic *Helicobacter pullorum* (Waldenström et al., 2003) and was isolated from diarrheic humans (Fox et al., 2000). However, no indications of impaired health were observed in treatments showing an enrichment of *H. canadensis* in the present study. Additionally, the mean relative abundance of *H. canadensis* was low (up to 0.68%). To our knowledge, no diet-induced alterations in the abundance of *H. canadensis* were reported in poultry. Nevertheless, Sun et al. (2022) and Hou et al. (2020) reported an increased abundance of closely related *H. pullorum* in the ceca of laying hens by supplementation of lignocellulose. However, causes and relevance of the increase in *H. canadensis* due to lignocellulose for poultry health and food safety remain unknown.

The carriers seemed to affect the site of AA absorption in the small intestine because treatment effects on AA digestibility differed between jejunum and ileum. Adding carriers increased the digestibility of 7 AA by the distal half of the jejunum and the digestibility of 13 AA by the distal half of the ileum, with bigger effects of LC than of SC on AA digestibility by the distal half of the ileum in most cases. However, the magnitude of effects on AA digestibility by the distal half of the ileum was low because the medians of increases were 1 and 2 %-units for SC and LC, respectively.

### Effects of the Silica-Based Carrier

The increase in nutrient digestibility following SC inclusion may be attributed to decreased ADFI. Lower ADFI was found to increase the retention time of the feed in the digestive tract (Castle and Castle, 1957). Post-prandial contractile activity was described to disperse digesta along the length of the small intestine, thereby increasing the access of enzymes to their substrates and maximizing contact area of digesta and the mucosa (Lentle and de Loubens, 2015). Therefore, lower ADFI when fed SC probably increased the access and time of enzymes to act upon their substrate. This is in accordance with Siegert et al. (2018), who observed increased AA digestibility for restrictive compared to *ad libitum* feeding.

The changes in the cecal microbiota due to the inclusion of SC might be explained by reduced substrate availability and higher digesta retention time. Reduced substrate availability upon feed restriction was assumed to cause changes in the cecal and fecal microbiota of broiler chickens (Siegerstetter et al., 2018; Metzler-Zebeli et al., 2019). In the present study, the higher nutrient digestibility observed for SC could have depleted the substrate available for microbial activity, thereby affecting the microbial composition. Further, higher digesta retention time due to lower ADFI upon addition of SC might have favored a more diverse microbiota, thereby increasing the α-diversity in the ceca. This is supported by Siegerstetter et al. (2017), who reported higher α-diversity in the ileum of male and in the ceca of female broiler chickens with decreased ADFI.

The reduced growth performance observed upon addition of SC in the present study does not imply that the carrier is unsuitable as an oil carrier in compound feeds. The carrier to oil ratio was identical for LC and SC and was defined by the capacity of LC to bind fluids. According to the SC manufacturer, the capacity of SC to bind oil was about 2.2 times higher than that of LC. Hence, SC probably absorbed moisture after ingestion due to its unused capacity to bind fluids, which may have contributed to reduced ADFI.

### Effects of the Rapeseed Oil Inclusion

There are several explanations for the higher digestibility of most FA and AA at 40 g/kg than at 20 g/kg oil inclusion. One explanation is a higher digestibility of the FA contained in the rapeseed oil compared to the basal diet. Such an effect has been described by Jørgensen et al. (2008), who reported increasing crude fat digestibility upon rapeseed oil addition of up to 160 g/kg of diet in broiler chickens. Further, the rapeseed oil contained more unsaturated FA and less saturated FA compared to the basal diet in the present study. As reviewed by Ravindran et al. (2016), the digestibility of saturated FA is increased when more unsaturated relative to saturated FA are fed because unsaturated FA support emulsification and formation of micelles of saturated FA. Such an effect may explain the higher digestibility of saturated FA at 40 g/kg than at 20 g/kg oil inclusion. Additionally, cis-unsaturated FA were described to increase the activity of pancreatic lipase (van Kuiken and Behnke, 1994). Therefore, the high content of C18:1, C18:2, and C18:3 in rapeseed oil might increase enzymatic fat digestion, resulting in higher digestibility of both unsaturated and saturated FA at the higher rapeseed oil inclusion. Inclusion of 40 g/kg compared to 20 g/kg oil also may have increased digesta retention time because an increased digesta retention time was described for the addition of soybean oil to diets for roosters (Yu et al., 2021). Such an effect may partly explain the increase in digestibility of CP and AA. However, the reduction in C16:0 digestibility at 40 g/kg compared to 20 g/kg oil inclusion in the present study cannot be explained.

Further research on the potential of rapeseed oil to modulate the gut microbiota in poultry is needed. Some mechanisms of how ingested fat may influence the gut microbiota have been reported. This includes antimicrobial effects of FA or bile acids (Mokkala et al., 2020). In addition, rapeseed oil contains other compounds with antimicrobial properties, such as phytosterols or ferulic acid (Shen et al., 2023). In the present study, the rapeseed oil inclusion had a bigger influence on the abundance of genera than adding the carriers (Figure 2). In particular genera of the families Lachnospiraceae and Oscillospiraceae were increased at the higher rapeseed oil inclusion when diets contained no carrier. Taxa of both families are able to produce butyrate (Vital et al., 2014), which might be beneficial for the epithelial integrity and immune development of the birds (reviewed by Maki et al., 2019). However, we are not aware of other studies investigating effects of rapeseed oil on the gut microbiota of poultry.

In conclusion, the results of the present study do not give an indication of an incomplete release of the oil from the carriers because both carriers did not reduce the TTR of most FA. Instead, digestibility or retention of most investigated nutrients increased upon carrier inclusion. For LC, effects on the cecal microbiota may have contributed to increased MEn. The decreased growth upon addition of SC might be attributed to unused capacity of SC to bind fluids, which may have led to moisture absorption in the digestive tract. The results suggest that LC can be used without drawbacks for nutrient digestibility and growth, while the use of SC warrants further examination with an exhausted capacity of SC to bind oil.

## Appendix Supplementary materials

Image, application 1
